# The Schott Treatment for Heart Disease

**Published:** 1895-10-19

**Authors:** Richard Greene

**Affiliations:** Medical Superintendent Northampton County Asylum, Berrywood.


### THE SCHOTT TREATMENT FOR HEART DISEASE.

By Richard Greene, F.R.C.P.E., Medical Superintendent Northampton County Asylum, Berrywood.

There is, unhappily, no need to enlarge on the extreme importance of the treatment of chronic diseases of the heart. When this organ, this wonderful organ throbbing in the very centre of the loom of life, fails, its failure brings in its train that long list of ills but too well known to us all. And these worry the physician not a little, for he feels his powerlessness to cure, too often to alleviate. Surely, therefore, a cordial welcome should await any discovery which will free the patient from his suffering and the physician from the opprobrium.

In the year 1882 Dr. Theodor Schott and his brother Dr. Adolph Schott, of Frankfort-on-the-Main, began a series of experiments on the effects produced on the blood pressure of lower animals by immersion in saline solutions. About the same time, similar investigations were being made by their countryman Beneke and by Jakob, of Cudowa, in Poland, but these experiments were carried out to the fullest extent by the brothers Schott. They all, however, satisfied themselves that various salts, especially the chlorides, had a marked effect in modifying the blood pressure. They believed that the more volatile salts had a similar power, but to a much smaller extent, and they further suspected that the skin might not be the non-absorbing organ it was supposed to be, and that these volatile substances might find access to the body through the pores. These experiments were the foundation of the Schott treatment of heart disease, for it was argued that if these solutions could control the blood pressure of lower animals in a state of health, there was no reason why similar effects should not be produced in diseased men.

Not far from Frankfort, and close to the extreme end of the Taunus range of hills, stands the little town of Nauheim, and here saline springs at a temperature of 85 to 95 deg. Fahrenheit issue from the ground in a stream, strong and apparently inexhaustible. The waters were very suitable to bring the Schott theories to a practical test. The composition of the Nauheim springs is extremely complex, but the chief constituents are chloride of sodium, chloride of calcium, chloride of potassium, and bicarbonate of calcium; and there are traces of manganese, iron, and zinc. The iron is in the form of phosphate, bicarbonate, and arseniate. The water is very highly charged with free carbonic acid.

After numerous trials, carefully watched and carefully conducted, Schott found that almost any form of heart disease was benefited by the treatment he elaborated. I have said *almost*, because it would manifestly be unsuitable for thoracic aneurism, and much care would have to be observed in treating cases of extensive aortic insufficiency. Also in cases complicated by arterial capillary fibrosis, and small granular kidney. Possibly, also, in extensive emphysema.

The form of cardiac disease which most readily yields to the treatment is undoubtedly simple dilatation. I have seen a very extensively dilated heart reduced to its normal size in a few weeks, and all the concomitant symptoms banished. In some of these cases there is distinct valvular insufficiency with a loud soft murmur, and with the reduction of the heart the murmur entirely disappears. An illustrative case of this was in Nauheim during my stay. He had been treated for dilatation with a mitral murmur four years since, and from the conclusion of the treatment to the present he has been in good health, and no murmur audible. There is an opposite condition to this, in which an obstructive murmur is developed as the heart is reduced. These cases are said to be much rarer than the former, and I did not meet with one.

Nothing is more remarkable than the rapidity with which the reduction in the size of the heart takes place. A single bath will sometimes lessen the area of dulness by one inch, and a single series of exercises even more than that. Of course there is a tendency to expansion again after the immediate action of the bath or gymnastics has passed off; but the expansion, as a rule, does not reach its former limit, and hence there is a gradual approach to the normal.

In dilatation coupled with valvular lesion a very marked improvement often follows. The dyspnœa, the anæmia, the dropsy, all disappear with a few weeks' treatment, and the patient is restored, if not to health, to at least a fresh lease of comfort. Almost no case is too serious or too far gone not to be susceptible of some amelioration, and even in the most unpromising cases of all, it is worth remembering that the improvement has been obtained without the aid of medicine, and should a relapse take place at a season when the Nauheim treatment cannot be followed, the exhibition of the suitable medicines will have a more decided effect from having been omitted in the interim.

While the Schott treatment would, as already stated, be manifestly unsuitable for large thoracic aneurisms, there is reason to believe from some cases of incipient aneurism treated by Dr. Bezly Thorne that even this fell disease may not be beyond reach if taken in time.

But little medicine is prescribed by Dr. Schott, and his treatment at Nauheim consists almost entirely of baths and gymnastics. First, as to the baths. It is usual to begin with the thermal brine bath. At first the temperature is about 32½ deg. Celsus, and the duration about eight minutes. The time is increased by one minute a day until the duration of the bath reaches about twenty minutes, and as the duration is gradually increased, so is the temperature gradually decreased until it gets about 28 to 30 deg. Celsus. After each group of three baths there is always a pause of one day, and sometimes it is desirable to have this pause after a bath has been taken daily for two days. As these baths are being proceeded with, the saline constituents are gradually increased by the addition of mutterlauge or mother liquor. At first one litre is added; then two and then three. The latter is not often exceeded. We have, therefore, three conditions present: first, an increasing duration of bath; second, a diminishing temperature; third, an increasing strength of salines.

After some twenty or twenty-five of the baths have been gone through, it is usual for the patient to be subjected to the sprudel baths. This water contains all the constituents of the thermal brine bath, but in the sprudel their solubility is perfect, from the presence of a large amount of carbonic acid, and the water is clear and sparkling, whereas it is reddish-brown colour in the thermal. The thermal is, indeed, the sprudel from which the carbonic acid has been allowed to escape. The sprudel baths are regulated in the same careful way as regards duration as are the thermal, but no mother-lye is added, nor is the temperature changed. It is the natural temperature of the water, that is, about 86 deg. Fahrenheit. These sprudel baths are very refreshing. The carbonic acid collects in minute globules on the surface of the body, and on leaving the bath a pleasant after-glow of warmth is experienced, owing to the direct stimulating effect which the water has on the skin. When fifteen or twenty of these baths have been taken, the patient is ordered the strom-sprudel. In this bath the water passing in at one aperture of the bath flows out at another after it has surrounded the patient with its foaming, eddying stream.

When taking any of these baths the patient should remain perfectly still, and after the bath he should rest in the horizontal position for an hour and a-half or two hours.

The effects of the baths—at least those perceptible to the patient—are rather curious. A strange sensation of oppression in the chest is often complained of. In me it amounted to a "girding" sensation, as if hoops were being applied to the thorax. I found this sensation most marked in the first half-dozen baths, and it became gradually fainter afterwards.

Now and then flying pains in the larger joints are felt. Occasionally, and in subjects not previously suspected of the gouty diathesis, attacks resembling those of acute gout are induced. This fact should be remembered, as it seems to prove that the action of the baths is not entirely due to the temperature of the water, nor the time the patient remains in it.

Then, if the heart has been carefully mapped out before the bath, and examined again afterwards, it will be found that it has diminished in size by an amount varying from half-an-inch to two inches; and the apex beat which had previously been diffuse and external to the nipple is now drawn upwards and inwards, and is more easily felt. The pulse varies considerably in different patients, and even in the same patient at different times; but the most common result is a reduction of four, or perhaps five or six, beats in a minute, and, what is of more importance, the volume is almost invariably increased. The irregular pulse of mitral disease is often made less irregular as well as stronger. I found this result in a young medical man who was under treatment during part of my stay.

Concurrently with the baths, resistance gymnastics are carried out. At the outset I should state that these exercises have nothing in common with what is implied by our English word "gymnastics." It is a misleading term to us. The movements are performed very slowly and deliberately. No mechanical appliances are used, as the resistance is offered by the hand of the operator. There are nineteen of these movements. The idea of regulated exercises in the treatment of chronic heart disease is by no means new. It is at least as old as the time of Stokes, and was referred to by him. But I believe I am right in saying that these Nauheim movements, this *widerstands-gymnastik*, was first introduced and elaborated by Schott, his object being to call into successive action every group of muscles in the body. Each movement takes about thirty or forty seconds, and there is a pause of like duration. The same movement is never performed twice in succession, but as the whole exercise has to last half an hour it is usual to repeat some of the movements on completion of the series. During the whole of the movements the breathing must be watched carefully, as there is a tendency on the part of some patients to hold their breath. In the leg movements one of the patient's hands should be placed on the back of a chair, but here again care must be taken that this support is in no way used for pulling or pressure, for if so the walls of the chest may be fixed and the beneficial effect of the movement diminished or lost.

The amount of resistance offered by the operator must never be great. I know that this word "great" conveys no accurate notion of the strength which ought to be used, but I am not aware that the force to be used has ever yet been put into foot-pounds. Indeed, it would necessarily vary with different patients, but it may be stated with the utmost confidence that the benefit derived is not in proportion to the force used, and that within certain limits, which experience will soon teach, the gentler the movements the better will be the results. Simple as these movements seem to the onlooker, their effects are little short of miracuous. In less than half an hour the dulness may be reduced by two inches, and the hepatic dulness decreased by a similar amount. In some very rare cases alarming syncope has been known to follow these gymnastics, and in certain patients it may be desirable to begin the treatment with baths and massage, reserving the exercises for a week or two.

It has been pointed out by Dr. Bezly Thorne that the heart, in its transition from extreme dilatation to the normal, passes through several distinct stages. At first the outline is semi-circular; it then becomes bipartite, and as the reduction goes on it assumes a conoid outline, and, finally, there is a tilting up of the right side, by which the normal is attained. One case which I followed at Nauheim satisfied me that Dr. Thorne's view was correct.

The season at Nauheim lasts from May 1st to October 1st. The time occupied in any individual case is from five to seven weeks. Then there is the "after-cure," which should be carried out among the hills, and which will be longer or shorter according to the time and money at the disposal of the patient. It should not be less than ten days or a fortnight.

What is the extent of improvement which may be looked for in chronic heart disease with valvular mischief? That is a difficult question to answer, because no two cases of heart disease are exactly alike. Suffice it to say, that apparently extreme cases, where any kind of occupation was impossible, have gone to Nauheim and have so far improved as to be able to resume their work, and that an annual visit to Nauheim has enabled them to maintain a fair standard of health. An equally difficult question would be the duration of the cure. I mean the length of time which the patient will retain moderate health. It is certain that cases have been under observation for many months, some for several years, and in many of these no return of the unpleasant symptoms has been seen. Of course, we must be prepared for occasional failure and relapse, as we have to deal with two unstable elements—the nature of heart disease and the self-restraint of the patient. If a man resume his evil ways as regards diet, smoking, want of proper rest, and so on, he has no right to complain if his symptoms return. Life must be carefully regulated for the cardiac sufferer.

During a month's residence at Nauheim I saw the treatment being carried out in a great many cases, and I closely followed it in a few. Indeed, I submitted to both baths and gymnastics until I had grasped the principles and practice of the Schott method. Nothing connected with the treatment struck me so forcibly as the extreme rapidity with which improvement took place. I have seen a patient so weak and anæmic from valvular disease and dilatation that he could not stand while the physical examination was being made, and I have seen that man within a fortnight walk about the town with perfect ease; the anxious expression gone and the anæmia and dyspnœa much reduced. And this was not an exceptional case. How are we to explain these results? It might be conceded, probably it must be conceded, that the action of the baths is chiefly mechanical—chiefly one of temperature and duration, although I am convinced, and what I have already said seems to prove it, that other influences are at work. It may be that the action of the gymnastics is one of suction only, drawing the blood from a distended heart to the muscles, and so giving the organ a chance of contracting and expelling all the blood from its cavities. But I offer no explanations. I leave the investigation of causes to the physiologist. Like Paul of Tarsus, I testify to those things which I have seen, and like another great man I explain nothing. To the patient it is a matter of indifference what has been at the root of his cure. He is satisfied that recovery or improvement has taken place.

This much is at present certain. The Schott treatment does exercise a decidedly beneficial effect on various cardiac diseases, explain the fact how you will. The most apparently hopeless cases of valvular trouble are often improved, and extensive dilatation has been cured at Nauheim over and over again. This is fortunately beyond controversy, and therefore this treatment has added to our armamentarium a new weapon wherewith to fight these chronic heart affections, which weapon can no longer be neglected by the profession. As yet, although thirteen years old, it is not much known in England. Very few practitioners have studied it sufficiently to give an intelligible account of it. Foremost among these few is Dr. Bezly Thorne, to whom, indeed, by his book, by his articles in the *Lancet*, and his papers at the British Medical Association, rests the merit of having introduced it to his medical brethren in England. Among others who have studied the subject in Nauheim are Dr. Frederic Thorne, of Leamington; Professor Leith and Professor Sir Grainger Stewart, of Edinburgh; Dr. Fisher, and Dr. Broadbent, of London. Sir Philip Smyly has published several cases in the *Dublin Medical Journal*. These names will show that an interest has been awakened among us, and I feel sure that only investigation is needed to confirm and extend its adoption.

Hundreds upon hundreds of cardiac sufferers who had regarded their cases as hopeless have after a six or seven weeks' course at Nauheim, returned so much better as to be able to resume their old places in the world, and for this they are indebted to the investigations of the Brothers Schott. It is not too much to say that mankind owes a debt of gratitude to these men which it will not easily repay. Our hearts will still beat our funeral marches to the grave, but those who have been afflicted with cardiac disease will find that a load has been taken off their shoulders, and that their march will be slower and easier.

As the Schott treatment becomes better known, and English sufferers invade Nauheim, the present bathing arrangements will be totally insufficient. Even now it is not always possible to obtain a bath at a reasonable hour without waiting an unreasonable time, and waiting is apt to induce irritability, too often present in cardiac cases without any external aid. Then there is neither hotel nor restaurant in Nauheim where English ideas of comfort or meal hours are studied. To many invalids this is a great drawback. A further objection is that many patients who would be benefited by the treatment cannot be induced to go abroad. There is the language difficulty which frightens many, and there are the expense and fatigue of a long journey.

All these objections can only be satisfactorily met in one way. That is, by providing means of carrying out the treatment in our own island. The waters of Nauheim can be imported in concentrated form, or they can be easily prepared sufficiently near for all practical purposes. In fact, this has been done in London, and I think also in Leamington. And then we have various medicinal springs, among others those of Leamington and Llangamarch Wells. For many generations the latter have had a local reputation in the treatment of heart disease, and lately baths have been fitten up, and an hotel opened. But at present all these are for the few. I want to see them for the many, and with an improved train service there could be no better place than Llangamarch. It is within easy distance of North Wales, and the Cumberland and Scotch hills are not far off. In any of these the *nach-kur* could be efficiently carried out.

Nevertheless, Nauheim can never be altogether superseded, at least as long as Theodor Schott lives. It was here the treatment was elaborated by him and his late brother. Here, too, the tired man is away from business cares and worries of every kind. The old troubles that had kept him down gradually fade away into the past, and peace overtakes him. As recovery approaches he dreams of the *nach-kur* amid the Swiss hills, and of a return to England, with body and mind recruited for his winter's work.

